# Nicorandil Ameliorates Doxorubicin-Induced Cardiotoxicity in Rats, as Evaluated by 7 T Cardiovascular Magnetic Resonance Imaging

**DOI:** 10.1007/s10557-021-07252-5

**Published:** 2021-09-30

**Authors:** Yixuan Wan, Bo He, Dongyong Zhu, Lei Wang, Ruijue Huang, Shiyu Wang, Chunhua Wang, Mengdi Zhang, Lu Ma, Fabao Gao

**Affiliations:** 1 Present Address: Department of Radiology, West China Hospital, Sichuan University, No. 37 Guoxue Road, Chengdu, 610041 China; 2 Molecular Imaging Center, West China Hospital, Sichuan University, Chengdu, China; 3 Basic Medical School, Yunnan University of Chinese Medicine, Kunming, China; 4 Department of Radiology, Huashan Hospital, Shanghai, China; 5 Sichuan Cancer Hospital and Institute, Sichuan Cancer Center, School of Medicine, Radiation Oncology Key Laboratory of Sichuan Province, University of Electronic Science and Technology of China, Shanghai, China

**Keywords:** Cardiotoxicity, Doxorubicin, Nicorandil, Cardiac magnetic resonance

## Abstract

**Purpose:**

Doxorubicin-induced cardiotoxicity (DIC) is a common side effect of doxorubicin chemotherapy, and a major mechanism of DIC is inflammation. However, no effective method exists to prevent DIC. In the present study, we investigated the cardioprotective effects of nicorandil against DIC using multiparametric cardiac magnetic resonance (CMR) imaging and elucidated the anti-inflammatory properties of nicorandil in rat models.

**Methods:**

Male Sprague-Dawley rats received four weekly intraperitoneal doxorubicin doses (4 mg/kg/injection) to establish the DIC model. After treatment with or without nicorandil (3 mg/kg/day) or diazoxide (10 mg/kg/day) orally, all the groups underwent weekly CMR examinations, including cardiac function and strain assessment and T2 mapping, for 6 weeks. Additionally, blood samples and hearts were collected to examine inflammation and histopathology.

**Results:**

According to our results, the earliest DIC CMR parameter in the doxorubicin group was T2 mapping time prolongation compared with the DIC rats treated with nicorandil (doxorubicin+nicorandil group) at week 2. Subsequently, the left ventricular ejection fraction (LVEF) and global peak systolic myocardial strain in the doxorubicin group were significantly reduced, and nicorandil effectively inhibited these effects at week 6. Our results were confirmed by histopathological evaluations. Furthermore, nicorandil treatment had a protective effect against the doxorubicin-induced inflammatory response. Interestingly, similar protective results were obtained using the K_ATP_ channel opener diazoxide.

**Conclusion:**

Collectively, our findings indicate that nicorandil application ameliorates DIC in rats with significantly higher cardiac function and myocardial strain and less fibrosis, apoptosis and inflammatory cytokine production. Nicorandil prevents T2 abnormalities in the early stages of DIC, showing a high clinical value for early nicorandil treatment in chemotherapy patients.

**Supplementary Information:**

The online version contains supplementary material available at 10.1007/s10557-021-07252-5.

## Introduction

Doxorubicin is a highly effective and frequently used chemotherapeutic agent [1]. However, cardiotoxicity and subsequent heart failure are fatal side effects of doxorubicin, limiting its clinical use. Depending on the cumulative dose, the incidence of doxorubicin-induced cardiotoxicity (DIC) is approximately 11–18% [2, 3]. However, to date, no effective treatment is available to counteract the progressive harmful action of doxorubicin and improve the prognosis of patients with DIC.

Nicorandil is an ATP-sensitive potassium channel (K_ATP_) opener [4] that has attracted keen interest because of its cardioprotective effect and because it improves left ventricular remodelling in rats with ischaemic heart failure [5]. Nicorandil alleviates apoptosis in diabetic cardiomyopathy through the PI3K/Akt pathway and combats DOX-induced nephrotoxicity by altering the TLR4/P38 MAPK/NF-κB signalling pathway [6, 7]. In a study of patients undergoing percutaneous coronary intervention, nicorandil suppressed the production of inflammatory cytokines such as interleukins (IL-1β and IL-8) and tumour necrosis factor-alpha (TNF-α) [8]. To date, the few studies investigating the effect of nicorandil on DIC have shown protective effects. However, neither cardiac magnetic resonance (CMR) studies nor inflammatory responses of nicorandil against DIC have been reported.

The early detection and treatment of cardiotoxicity are critical to recover cardiac function and reduce the incidence of associated adverse cardiac events [9, 10]. The current methods to identify the early stages of DIC are limited. The current clinical diagnostic standard is left ventricular ejection fraction (LVEF) measurement [3, 11]. However, LVEF values are usually within the normal range when irreversible myocardium damage occurs [12, 13]. The lack of a validated early damage marker limits the development of preventive strategies and drug therapy. Both preclinical and clinical studies have suggested the potential for CMR multiparametric analysis to detect acute DIC [14–16], and CMR allows accurate characterization of myocardial tissue [13, 17, 18]. Thus, CMR is suitable to detect myocardial oedema and strain, which are present at different stages of DIC [18–21]. Accordingly, this study aimed to perform early and continuous monitoring of the therapeutic effects of nicorandil on DIC in rats by CMR and identify early myocardial changes. We also investigated inflammatory activities and pathological correlations.

## Materials and Methods

### Study Design

**Fig. 1:**
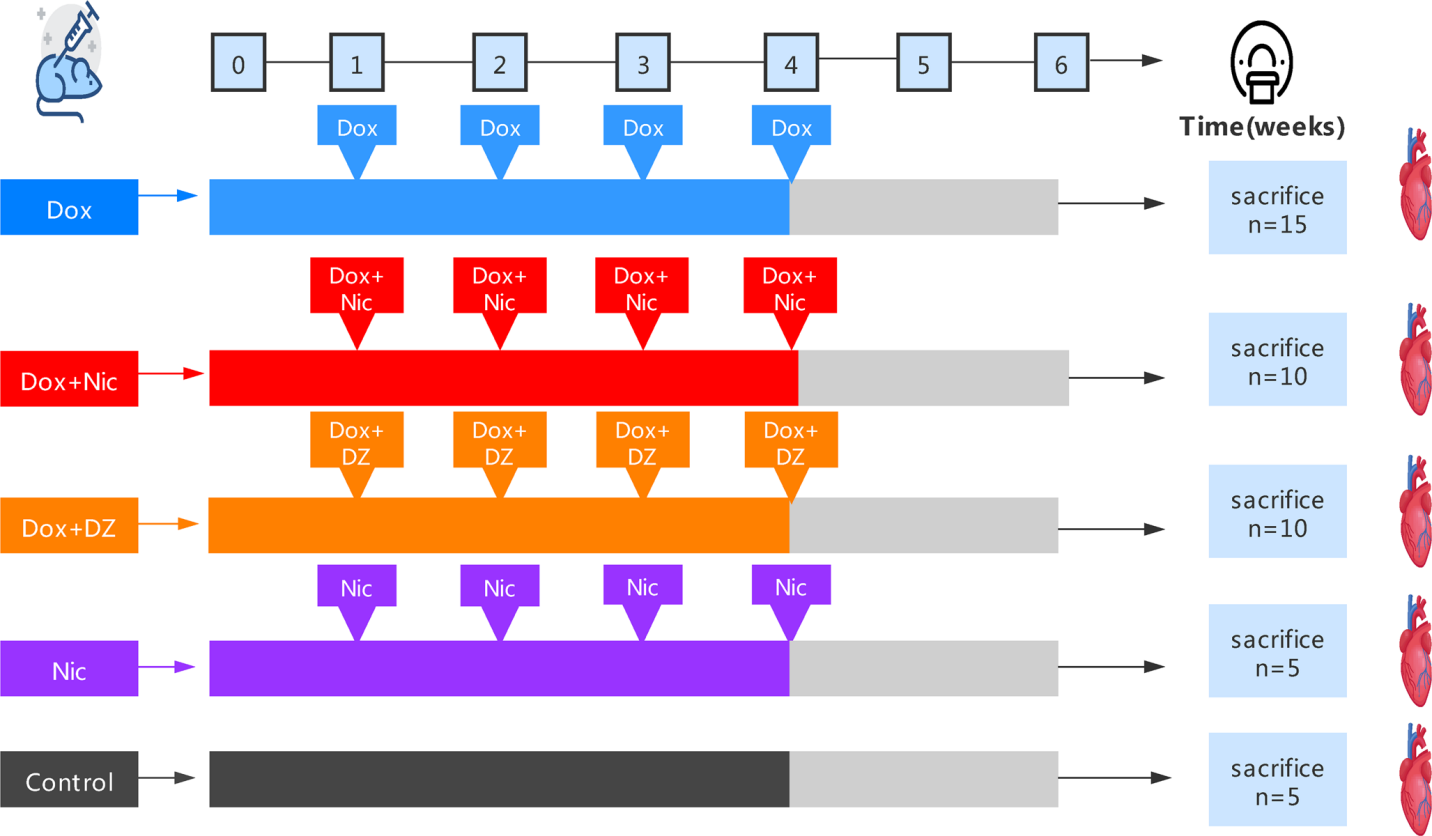
Study design

The use of animals in this study was reviewed and approved by the Institutional Ethics and Animal Care Committee of West China Hospital, Sichuan University. The study design is summarized in Fig. 1. Forty-five male Sprague-Dawley (SD) rats (175 g±) were randomized to five groups: (1) Dox group: 15 received four weekly doxorubicin injections (doxorubicin; D8740; Solarbio, Beijing, China) (to obtain a cumulative dose of 16 mg/kg, i.p.); (2) Dox + Nic group: ten rats received nicorandil (nicorandil; M0102A; Meilun, Dalian, China) (3 mg/kg/day, i.g.) and doxorubicin as in the Dox group for four weeks. The dose of nicorandil used was selected from a previous study [22]; (3) Dox + DZ group: ten rats received diazoxide (10 mg/kg/day, i.g.) and doxorubicin as in the Dox group for four weeks. The dose of diazoxide used was selected from previous studies [23–25]; (4) Nic group: five rats received nicorandil (3 mg/kg/day, i.g.) for four weeks; (5) Control group: five rats served as controls. All groups were followed until week 6, after which the rats were euthanized via intravenous injection of pentobarbital sodium.

### Cardiac Magnetic Resonance Imaging

All rats were imaged weekly using a 7.0-T small animal preclinical system (BioSpec 70/30; Bruker, Ettlingen, Germany). The magnetic resonance imaging system was equipped with a dedicated rat cardiac coil. The preparation work before scanning and specific operating procedures were performed as previously described [26]. The CMR protocol included a cardiac fast low-angle shot (FLASH) cine sequence to provide high-quality 2,4-chamber long-axis and short-axis stack views of the left ventricle (LV), a T2 mapping sequence, and late gadolinium enhancement (LGE) sequences. The imaging parameters of the cardiac FLASH cine sequence of the LV were as follows: flip angle (FA), 15°; field of view (FOV), 50 × 50 mm; repetition time/echo time (TR/TE), 8/3 ms; slice gap, 1.5 mm; slice thickness, 1.5 mm; matrix size, 256 × 256; read resolution, 0.26 × 0.26 mm/pixel; number of slices, 6–8. For T2 mapping based on the cardiac FLASH cine sequence using the same location as the cine images, the scanning parameters were as follows: repetition time/echo time (TR/TE), 1500/30 ms; number of slices, 6–8; slice gap, 1.5 mm; slice thickness, 1.5 mm; MTX, 192 × 192; field of view (FOV), 50 × 50 mm. The LGE images were acquired 6–8 min after a tail vein injection of 0.25 mmol/kg of dimeglumine gadopentetate. The imaging parameters were the same as the steady precession (FISP) technique, and the scanning parameters were as follows: FV, 25°; TR/TE, 8/3 ms. The scanning parameters for the fast imaging using the steady precession (FISP) technique were TR/TE, 5.2 ms/1.8 ms; flip angle, 25°; slice gap, 1.5 mm; slice thickness, 1.5 mm; matrix size, 256 × 256; read resolution, 0.26 × 0.26 mm/pixel; number of slices: 6–8.

### Image Analysis

For the analysis, the image was reconstructed in 2,4-chamber long-axis and short-axis views with a slice thickness of 1.5 mm using cardiac image analysis software (cvi 42; Calgary, Alberta, Canada). The cardiac function parameters, including the left ventricular myocardial mass (LVM), left ventricular end-systolic volume (LVESV), left ventricular end-diastolic volume (LVEDV) and LVEF, were calculated using semiautomated endocardial and epicardial contour tracing based on a short-axis cine stack. The left ventricular global peak longitudinal strain (GLS), left ventricular global peak radial strain (GRS) and left ventricular global peak circumferential strain (GCS) were semiautomatically tracked based on 2- and 4-chamber long-axis cine images and short-axis cine images. The specific operation was also manually drawn on the short axis and long axis of the cardiac cavity. T2 mapping values were automatically generated by drawing the endocardium and epicardium border. LGE was analysed by visual determination with no visible artefact [27].

### Plasma Biochemistry

Blood samples were collected into 3-ml heparinized tubes and then were centrifuged at 3000 rpm for 15 min to obtain plasma. The plasma levels of creatine kinase isoenzyme (CK-MB), creatine kinase (CK), lactate dehydrogenase (LDH), interleukins IL-1β and IL-18, and tumour necrosis factor-alpha (TNF-α) were detected using chemical kits (Sigma, Aldrich, Milan, Italy) according to the manufacturer’s instructions.

### Histopathology

Rats were euthanized via pentobarbital sodium injection after CMR at 6 weeks. Each heart was extracted immediately and fixed in a 4% paraformaldehyde solution. After fixation for 48 h, the heart was serially sectioned along the short-axis plane. Haematoxylin and eosin (H&E) staining was used to pathologically assess cardiac tissue. Masson’s trichrome staining was used to visualize the development of cardiac tissue fibrosis, which was subsequently examined by a pathologist (H.S. P.) blinded to the MR results.

### TUNEL Staining

Cardiac apoptosis was detected using terminal deoxynucleotidyl transferase-mediated dUTP nick-end labelling (TUNEL) and counterstainedusing 4′,6-diamidino-2-phenylindole (DAPI). The percent apoptosis was calculated by dividing the number of TUNEL-positive cells by the total number of cardiac cells viewed in the section.

### Immunohistochemical (IHC) Staining

Paraffin-embedded cardiac tissue sections were routinely prepared and then deparaffinized. Next, the sections were incubated with anti-NLRP3 antibody (1:100 dilution; AdipoGen; San Diego, USA) at 4 °C for 15 h. Subsequently, the sections were incubated with secondary antibody at 37 °C for 30 min. The proteins were stained with 3,30-diaminobenzidine (DAB) for 1 min and counterstained with haematoxylin. Finally, the stained sections were photographed under a microscope.

### Statistical Analysis

The variables are expressed as means ± SD or medians as appropriate. The normality of the data distribution was assessed using the Shapiro–Wilk test, and the homogeneity of variance was assessed using Levene’s test. One-way ANOVA with the LSD post hoc test was used for multiple groups, and two-way repeated measures ANOVA with Bonferroni correction was used to analyse the changes in the imaging variables across time. Differences were considered statistically significant at *p* < 0.05. All the data were analysed using SPSS Statistics version 27.0 (IBM), and graphics were created using GraphPad Prism software 8.0 (GraphPad Software, La Jolla, USA).

## Results

### Nicorandil Prevents Doxorubicin-Induced Body Weight and Mortality Rate

**Fig. 2:**
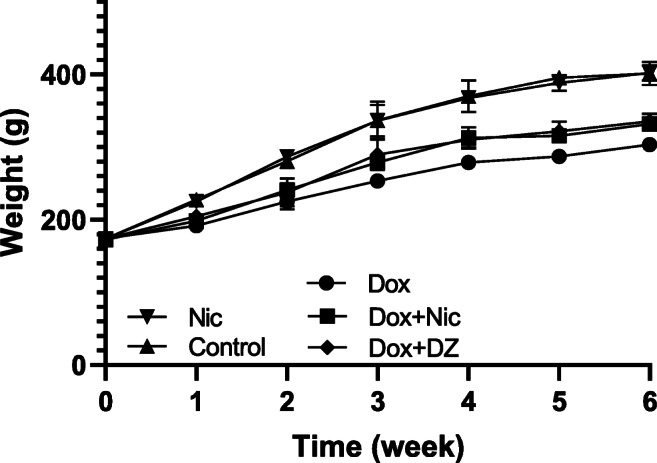
Body weight of the rats in the control, Nic, Dox, Dox + Nic, and Dox + DZ groups at different time points. The data are expressed as means ± SD

The body weights of the rats in all groups increased steadily over the 6 weeks of the experiment. However, compared with those in the control group, the body weights of the rats in the Dox group were reduced at each time point. Coadministration of doxorubicin with nicorandil or diazoxide prevented the effect of doxorubicin on body weight (Fig. 2). At 6 weeks, in the Dox group (*n* = 15), 40% (7/15) of the doxorubicin-treated animals died during the experiment. However, in the control group (*n* = 5), the Dox + Nic group (*n* = 10), and the Dox + DZ group (n = 10), no rats died, with a survival rate of 100% (Table 1).

**Table 1:** Body weight, heart weight and survival rate of rats in the five groups at week 6

Group	Mortality (%)	FN	HW (g)	Initial BW (g)	Final BW (g)	HW/BW ratio (×10^−3^)
**Control**	0	5	1.3 ± 0.06	174.5 ± 4.07	401.5 ± 9.99	3.24
**Nic**	0	5	1.32 ± 0.07	174 ± 3.32	402.29 ± 10.26	3.28
**Dox**	47	8	1.02 ± 0.15^***^	174.4 ± 3.96	303.63 ± 3.78^***^	3.36
**Dox + Nic**	0	10	1.06 ± 0.05^***^	172.3 ± 3.54	332 ± 5.7^***###^	3.19
**Dox + DZ**	0	10	1.07 ± 0.03^***^	173.7 ± 2.18	334.67 ± 10.34^***###^	3.2

^***^p < 0.001 compared with the control; ^**###**^*p* < 0.001 compared with the Dox group. FN, final number; HW, heart weight; BW, body weight;

### Nicorandil Attenuates Doxorubicin-Induced Cardiac Injury

**Fig. 3:**
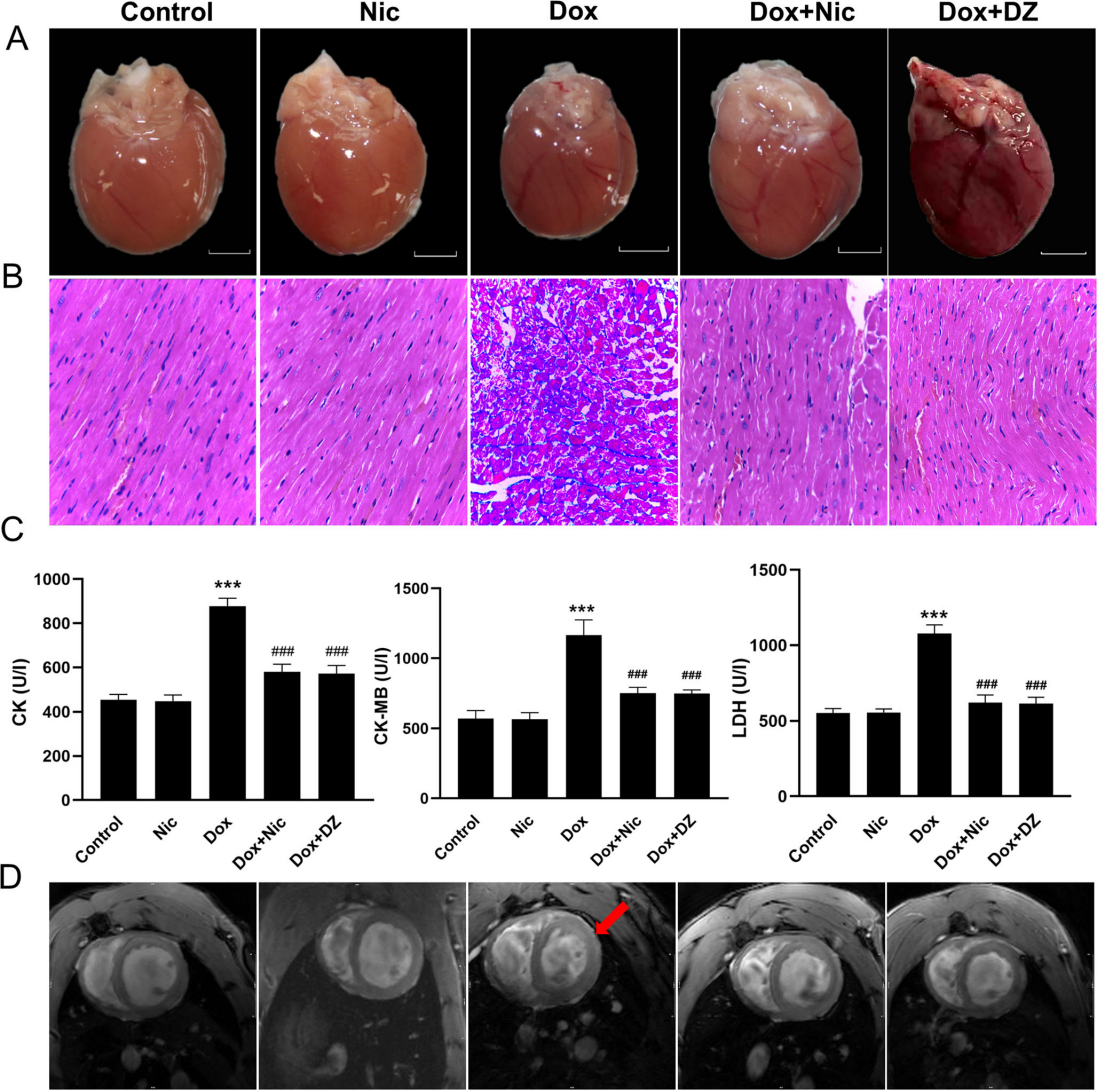
Effect of nicorandil on doxorubicin-induced cardiac injury. (**A**) Representative gross images of the whole heart. (**B**) Representative images of HE staining in the left ventricles (LV) of the five groups (×400) (*n* = 5–8). (**C**) CK, CK-MB, and LDH levels in the five groups measured by ELISA (n = 5–8). (**D**) Late gadolinium enhancement (LGE) images (red arrow, hyperintense). The data are expressed as means ± SD. ^***^p < 0.001 versus control. ^**###**^*p* < 0.001 versus Dox

The heart size was reduced in the Dox group compared with that in the control group, but the heart size in the Dox + Nic group was larger than that in the Dox group (Fig. 3A). Additionally, compared with that in the control group, H&E staining of heart sections in the Dox group revealed myocardial injury with cardiomyocyte vacuolization and myofibril loss, which were reduced in the Dox + Nic group (Fig. 3B). The plasma levels of CK, CK-MB, and LDH, which are markers of cardiac injury, were measured. The plasma CK, CK-MB, and LDH levels in the Dox group were significantly increased compared with those in the control group; by contrast, the levels in the Dox + Nic group were significantly lower than those in the Dox group (Fig. 3C). LGE images showed no visible intensive foci in the control group or Dox + Nic group, whereas typical inflammatory/necrotic lesions were observed in the Dox group (Fig. 3D). These results suggest that nicorandil exerts cardioprotective effects against DIC. Interestingly, diazoxide showed the same protective effect as nicorandil against DIC.

### Nicorandil Prevents Doxorubicin-Induced Cardiac Dysfunction

**Fig. 4:**
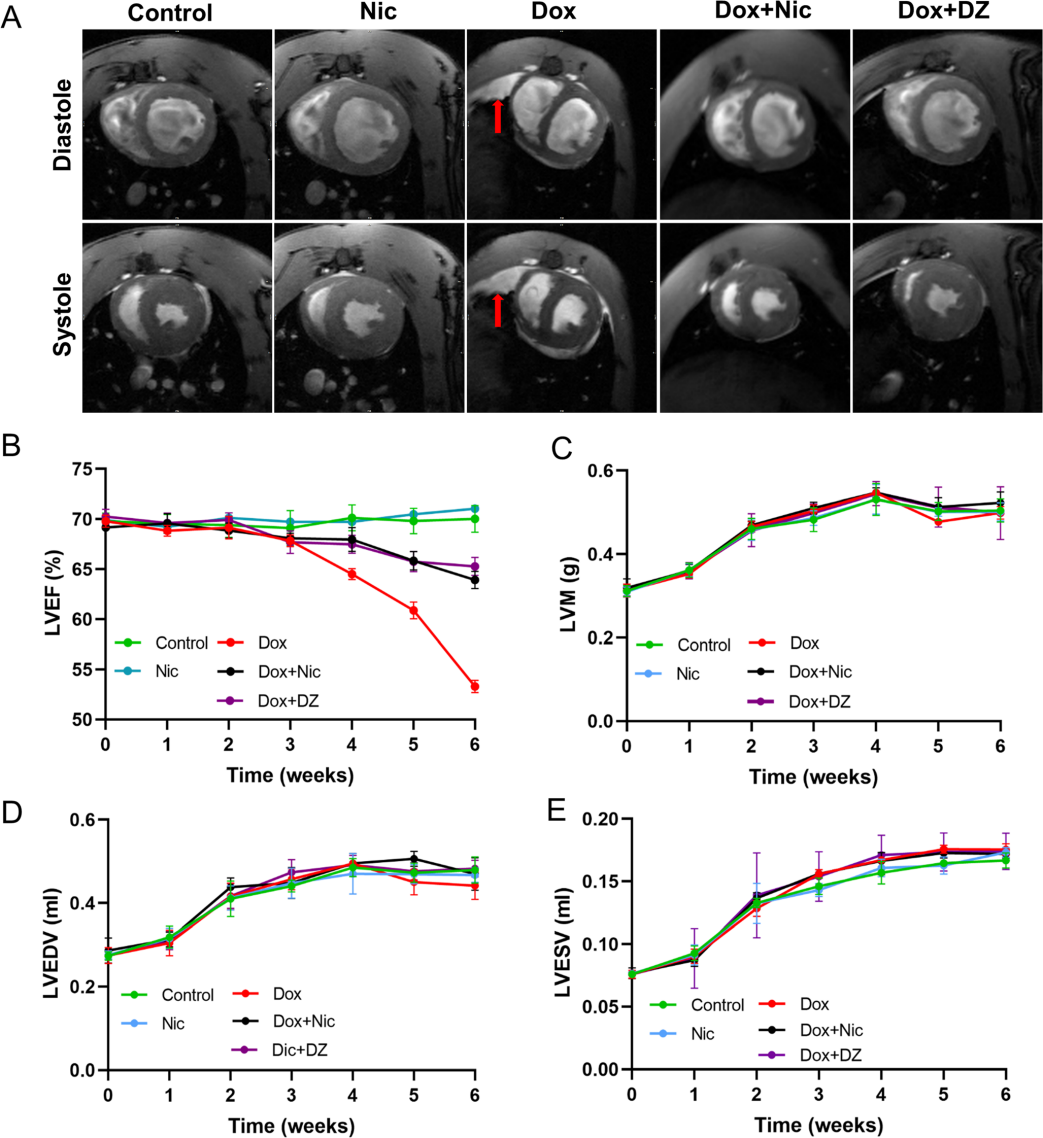
Nicorandil treatment improves cardiac function. (**A**) Representative diastolic and systolic CMR images of the left ventricles from five groups at week 6 (*n* = 5–8) (red arrow, pericardial effusion). (**B**) Progression of the LVEF during the protocol (n = 5–8). (**C**–**E**) Progression of the LVM (C), LVEDV (**D**) and LVESV (**E**) during the protocol (n = 5–8). The data are expressed as means ± SD. LVEF, left ventricular ejection fraction; LVM, left ventricular mass; LVEDV, left ventricular end-diastolic volume; LVESV, left ventricular end-systolic volume

Representative CMR images are shown in Fig. 4A, and weekly CMR examinations revealed that compared with that in the control group, the LVEF in the Dox group remained unchanged until week 3. Subsequently, the LVEF progressively deteriorated until the end of the study, and the LVEF in the first three weeks was significantly higher than that at weeks 4 to 6 (Supplemental table; Fig. 4B). The LVEF was significantly lower in the Dox group than in the Dox + Nic or Dox + DZ group at weeks 4 to 6 (all *p* < 0.05) (Supplemental Table; Fig. 4B). Similarly, compared with the control group, the LVM and LVEDV in the Dox group were significantly decreased at weeks 4, 5 and 6 (all *p* < 0.05) but were significantly increased in the Dox + Nic and Dox + DZ group (Fig. 4C, D). Moreover, no significant difference was found in the LVESV among the five groups at any timepoints (Fig. 4E).

### Nicorandil Reduces Myocardial Oedema Associated with DIC

**Fig. 5:**
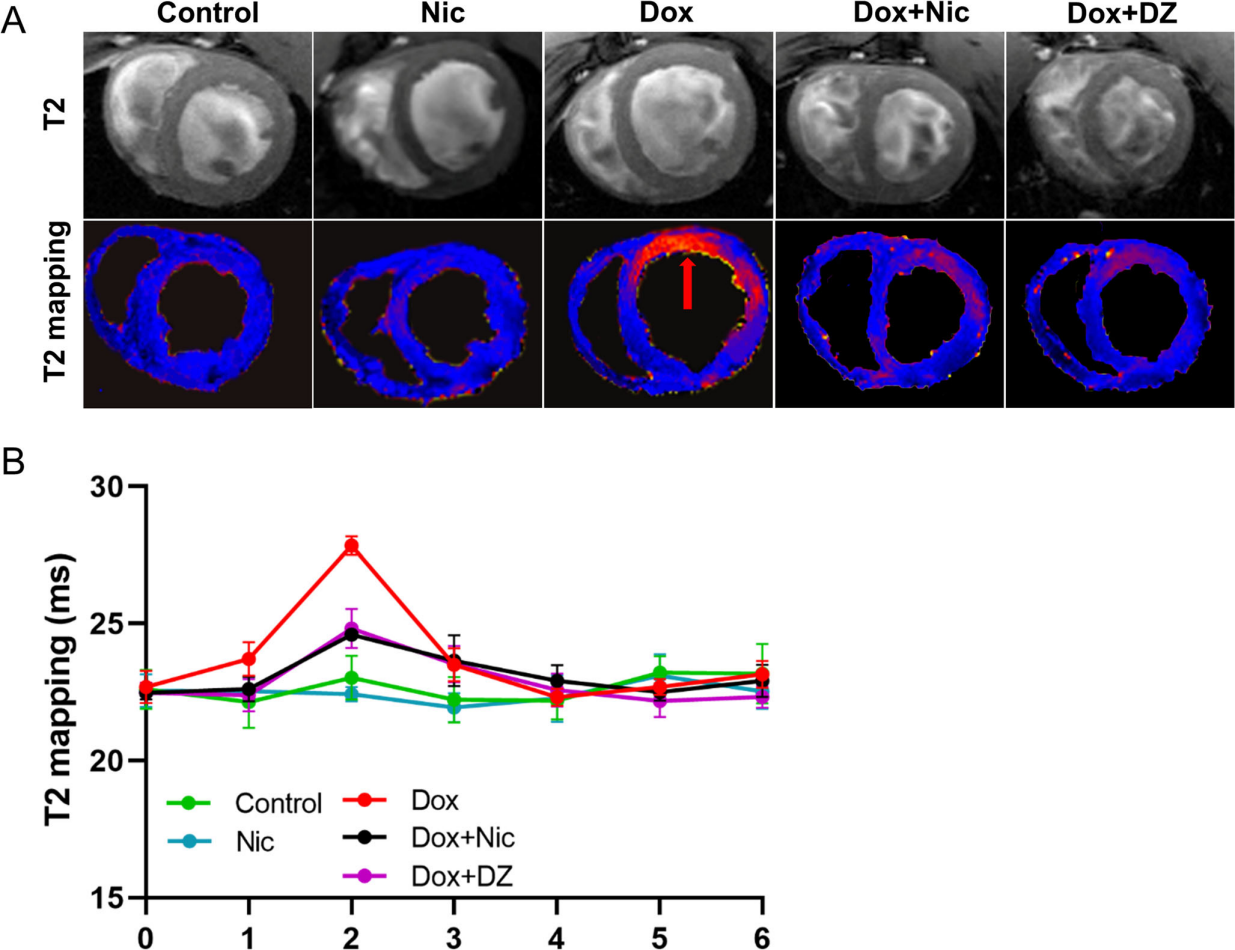
Nicorandil treatment reduces myocardial oedema. (**A**) Representative images of T2 mapping in the five groups at week 2 (*n* = 5–8) (red arrow, hyperintense). (**B**) Progression of T2 mapping during the protocol (n = 5–8). The data are expressed as means ± SD

Representative T2 images are shown in Fig. 5A, and weekly CMR examinations revealed that T2 values were significantly elevated in doxorubicin-treated rats after their second dose and tended to decrease after their third dose. The T2 values in week 2 were significantly higher than those in the other weeks (all *p* < 0.001) (Fig. 5B) (Supplemental Table), which demonstrates that myocardial oedema peaked at week 2 after doxorubicin treatment. Additionally, the increase in T2 relaxation time was significantly lower in the Dox + Nic and Dox + DZ groups than in the Dox group at week 2 (all *p* < 0.001), while no significant difference was found at the other time points (Fig. 5B; Supplemental Table).

### Nicorandil Prevents Cardiac Strain in DIC

**Fig. 6:**
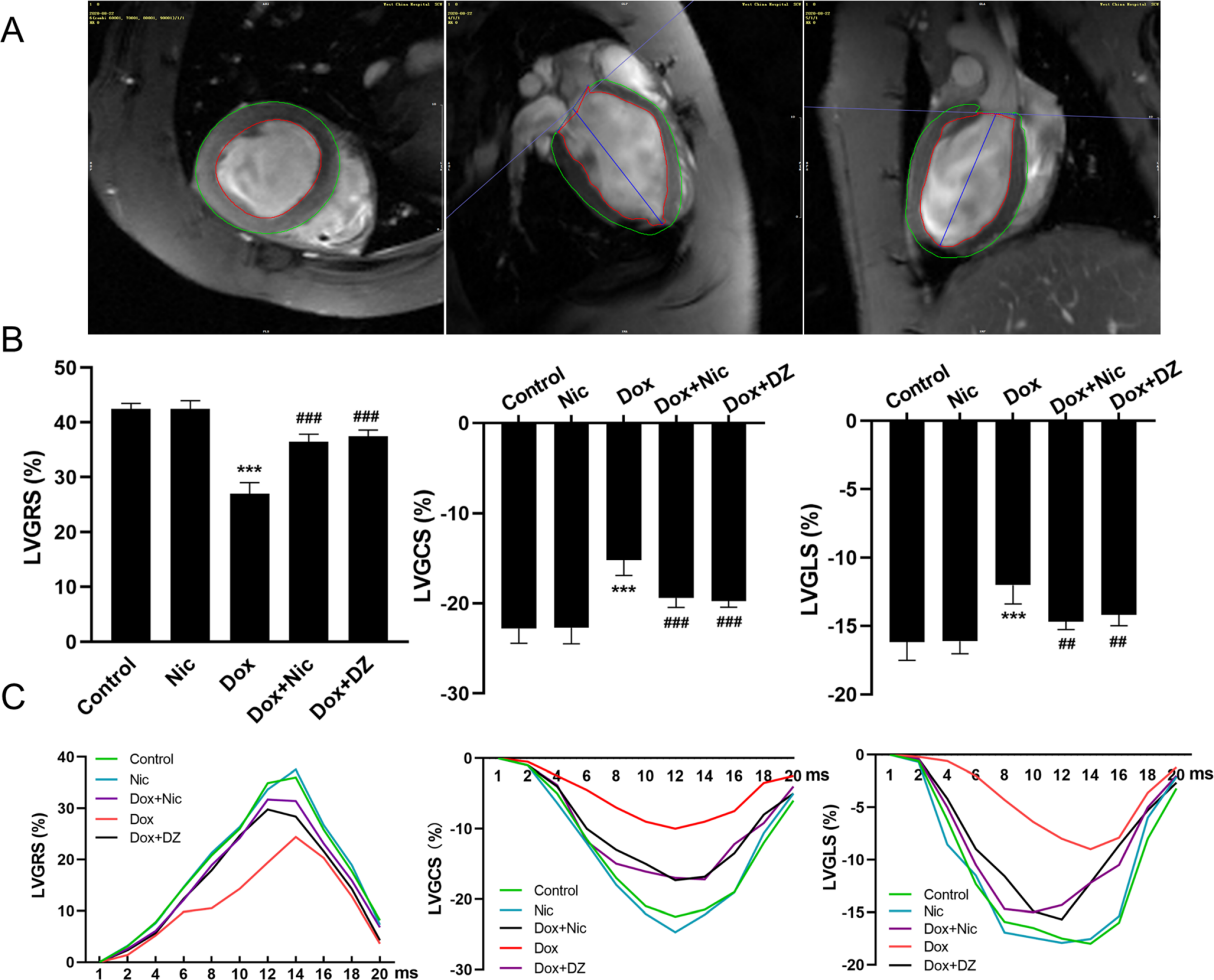
Nicorandil treatment improves cardiac strain. (**A**) Representative short-axis strain and 2,4-chamber long-axis strain images. (**B**) GLS, GRS and GCS at 6 weeks (n = 5–8) (**C**) Representative strain time curves of the five groups at 6 weeks. GLS, global peak longitudinal strain; GRS, global peak radial strain; GCS, global peak circumferential strain; LV, left ventricle. The data are expressed as means ± SD. ^***^p < 0.001 versus control. ^**##**^*p* < 0.01, ^**###**^*p* < 0.001 versus Dox

CMR tissue tracking images are shown in Fig. 6A. CMR examinations revealed that LV GRS, GCS and GLS were significantly decreased in the Dox group at 6 weeks compared with that in the control group (all *p* < 0.001). Nicorandil or diazoxide treatment increased these values (all *p* < 0.001) (Fig. 6B). The strain time curves in a cardiac cycle showed that strain was reduced in the Dox groups and that this effect was significantly suppressed in the Dox + Nic and Dox + DZ groups (Fig. 6C).

### Nicorandil Attenuates Apoptosis and Fibrosis during DIC

**Fig. 7:**
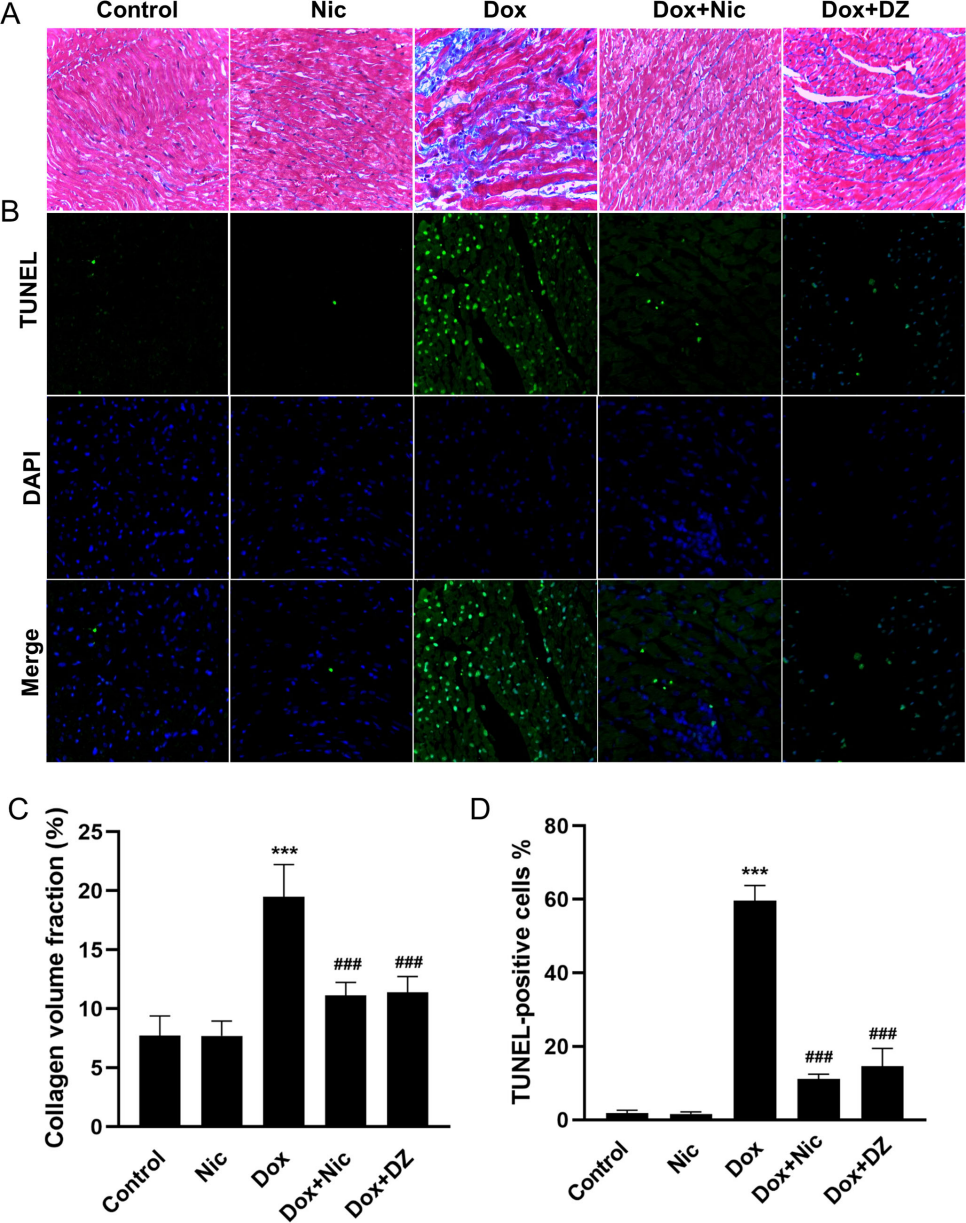
Effects of nicorandil on myocardial fibrosis and apoptosis. (**A**) Representative Masson’s trichrome staining images illustrating myocardial interstitial fibrosis at 6 weeks (×400) (*n* = 5–8). (**B**) Cell apoptosis as determined by the TUNEL assay (n = 5): apoptotic cells stained green; nuclei stained blue with DAPI. (**C**) Quantification of the relative fibrotic area by Masson’s trichrome staining (*n* = 5–8). (**D**) The apoptosis rate was determined by the TUNEL assay (n = 5). The data are expressed as means ± SD. ^***^p < 0.001 versus control. ^###^*p* < 0.001 versus Dox

Masson’s trichrome staining results showed that the degree of myocardial fibrosis was significantly increased in the Dox group compared with that in the control group and was clearly reduced by nicorandil or diazoxide treatment (Fig. 7A). The median LV collagen volume fractions (CVFs) in the Dox group were significantly higher than those in the control group, and treatment with either nicorandil or diazoxide abolished this effect (Fig. 7C). The number of TUNEL-positive apoptotic cells was significantly larger in the cardiac tissue of the Dox group rats than in that of control group rats, and cell apoptosis was prevented by nicorandil or diazoxide treatment (Fig. 7B, D).

### Nicorandil Attenuates the Doxorubicin-Mediated Inflammatory Response

**Fig. 8:**
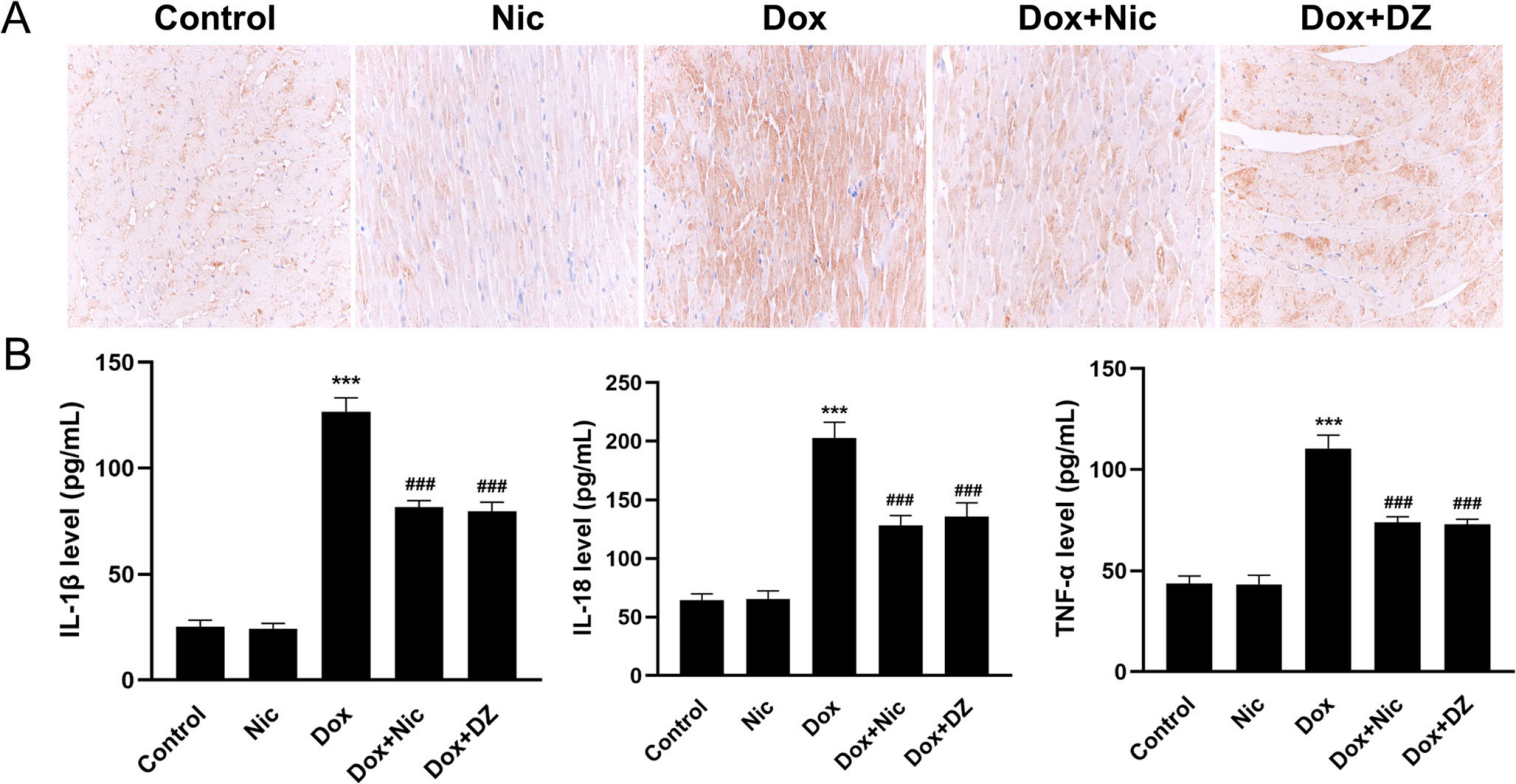
Nicorandil treatment attenuates the inflammatory response. (**A**) NLRP3 expression in the five groups as visualized by IHC (×400) (n = 5). (**B**) IL-1β, IL-18 and TNF-α levels in the five groups as measured by ELISA (n = 5). The data are expressed as means ± SD. ^***^p < 0.001 versus control. ^###^*p* < 0.001 versus Dox

We next examined the effects of nicorandil on the inflammatory response induced by doxorubicin in rats. IHC staining showed that compared with that in the control group, the protein expression of NLRP3 was markedly increased in the Dox group, but nicorandil inhibited this expression (Fig. 8A). Additionally, ELISA showed that the plasma levels of IL-1β, IL-18 and TNF-α were markedly higher in the Dox group than in the control group and that upregulation was remarkably attenuated by nicorandil (Fig. 8B). Furthermore, similar results were obtained in the Dox + DZ group.

## Discussion

In the present study, we demonstrated the cardioprotective effect of nicorandil in a rat model of DIC. Serial multiparametric CMR evaluation showed that nicorandil significantly attenuated cardiac dysfunction, global strain reduction and markers of myocardial injury associated with DIC. Hearts from the Dox + Nic group showed significantly less myocardial fibrosis and cardiomyocyte apoptosis and a significantly reduced inflammatory response. Furthermore, doxorubicin caused significant T2 prolongation after administration: at subclinical DIC stages (before overt cardiac dysfunction), the increase in the T2 relaxation time was significantly smaller in the Dox + Nic group. These findings demonstrate the beneficial effects of nicorandil in a DIC rat model regarding CMR.

Doxorubicin, a DNA topoisomerase II inhibitor, has been used clinically for more than 50 years and remains the first-line therapy for many cancer types [28]. Approximately 32% of all breast cancer patients, 60% of elderly lymphoma patients and most soft tissue sarcoma patients receive doxorubicin during their oncological treatment [29]. DIC is a frequent cardiotoxic side effect of doxorubicin. Depending on the cumulative dose of doxorubicin, the incidence of severe DIC, defined as a reduction in LVEF >10%, resulted in overt systolic heart failure that can be as high as 30% [30]. Severe DIC represents a considerable clinical challenge and a heavy burden on individuals and society.

The current clinical approach for DIC includes the early detection of LVEF and use of nonspecific heart failure (HF) therapies such as beta-blocker inhibitors [11, 31]. However, these changes reflect an advanced stage of myocardial damage. Almost 90% of patients developing doxorubicin-mediated LVEF deterioration never fully recover complete cardiac function even with these therapies. Nicorandil, the first nitrate compound and ATP-dependent potassium (K^+^ATP) channel opener applied clinically as a new vasodilator treatment [32, 33], has multiple cardiovascular benefits for multiple heart diseases and improved cardiac function [34–36]. Nicorandil suppresses the inflammatory cytokines IL-1β, IL-1, IL-6, IL-10, IL-18, IL-19 and TNF-α in acute coronary syndrome patients [37]. Su et al. further clarified that nicorandil effectively inhibits myocardial inflammation and ameliorates myocardial injury after coronary microembolization by inhibiting TLR4/MyD88/NF-κB signalling [38]. However, nicorandil is rarely tested in the context of DIC. Mari et al. [39] revealed in the HL-1 cardiomyocyte cell line derived from mouse atria that the mitochondrion was the target organelle of nicorandil and protected cardiomyocytes from doxorubicin-induced reactive oxygen species (ROS). Importantly, Ihab et al. [40] published the first in vivo evidence of the cardioprotection afforded by nicorandil in a rat model of DIC. Supporting the in vitro data, their results showed that cardiomyocytes subjected to nicorandil are protected against DIC. In a subsequent study, Lamiaa et al. [41] found that nicorandil is associated with improved haemodynamic perturbations, mitochondrial dysfunction and ultrastructural changes. Additionally, previous work has indicated that doxorubicin causes direct vascular injury, resulting in increased vascular tone and augmented arterial stiffness, which can predict DIC [42], but nicorandil protects vessels from severe doxorubicin toxicity by increasing NO availability, which reverses most of the cardiotoxicity caused by doxorubicin [40]. In our study, for the first time, we verified the protective effect of nicorandil on DIC from the perspective of cardiac function, myocardial strain and myocardial oedema through CMR methods. Notably, we found that LVEDV decreased and LVESV showed no significant change in the doxorubicin group compared with others, similar to a prior study [26]; however, a prior study reported a constant LVEDV and increase in LVESV in a cohort of cancer patients treated with doxorubicin [43]. Additionally, another animal study reported that both LVEDV and LVESV were significantly increased postchemotherapy [44]. The differences in results may be attributed to the changes in body weight, subject species, sample size, dosing factor or treatment regimens.

Although accumulating evidence indicates that iron metabolism, calcium disorders, topoisomerase inhibition, sarcomere disruption, mitochondrial damage, oxidative stress, and apoptosis underlie the toxicity of doxorubicin [45–47], most studies support the view that inflammation plays a key role in the pathogenesis of DIC. Doxorubicin has been shown to upregulate several inflammatory factors, such as IL-1β, IL-6, IL-8, IL-10, IL-17 and TNF-α [8, 48]. Doxorubicin therapy also increases NLRP3 inflammasome expression, promoting cardiac damage. Additionally, targeting the NLRP3 inflammasome can alleviate DIC [49, 50]. Here, we discovered that doxorubicin upregulates the expression of NLRP3, IL-1β, IL-18, and TNF-α and that nicorandil treatment reduces the expression of these inflammatory factors.

Our study provides the first demonstration that nicorandil protects the heart against DIC in a rat animal model based on CMR. In our model, doxorubicin has a clear cardiocytotoxic effect, and we can detect differences in cardiac function and cardiac strain between animals receiving nicorandil or no treatment. We also found differences in T2 mapping values implicated in DIC [19]. At the end of the study protocol, we found that nicorandil-treated animals had significantly less fibrosis (collagen fraction), apoptosis and inflammation than animals that received no pretreatment.

T2 mapping is an accurate technique to detect and quantify myocardial oedema [51]. We have previously used T2 mapping to characterize the oedematous reaction of porcine [19, 51, 52] and human [53] myocardium to ischaemia/reperfusion. T2 relaxation time prolongation correlates with an increased myocardial fluid content [51]. Our analysis demonstrates that the T2 relaxation times were increased in rats treated with doxorubicin at 2 weeks but returned to near baseline levels at other weeks, which might be responsible for the timing of imaging. DIC is a dynamic process and comprises several phases, and different pathological stages may have different imaging findings. Previous studies have reported that myocardial oedema precedes fibrosis in DIC and is in flux [18, 54, 55], likely explaining why T2 only observed anomalies at week 2. In our study, T2 mapping abnormalities provided the earliest marker of subtle myocardial damage, with prolongation of T2 relaxation times occurring before LVEF abnormalities were detected. These findings demonstrate that T2 relaxation-time prolongation identifies intracardiomyocyte oedema as the earliest DIC event and that early treatment with nicorandil significantly reduces myocardial oedema in the early stages of DIC.

In conclusion, our study supports previous findings [40, 41] on the efficacy of nicorandil against DIC. Importantly, our study is the first to demonstrate the protective effect of nicorandil against DIC with serial CMR evaluations, and this cardioprotective effect is accompanied by a reduction in inflammation levels. Additionally, we obtained similar results with the K_ATP_ channel opener diazoxide. Our results further demonstrate that T2 relaxation time increases at an early disease stage and that early administration of nicorandil is effective in reducing myocardial oedema and subsequent myocardial dysfunction, indicating that nicorandil is a promising cardio-oncological drug to prevent DIC in clinical applications.

## Study Limitations

One potential limitation of the present study is the intraperitoneal doxorubicin administration route, in contrast to the intravenous route used in cancer patients. A further limitation in this study is the use of healthy rats, which are free of the comorbidities that are common in cancer patients who develop DIC, many of whom are elderly individuals. Additionally, this study only comprised a few animals, which may limit the statistical power of the heart weight to body weight ratios.

## Supplementary Information

ESM 1 Supplemental table. CMR in vivo data for all the time points represented by groups as means ± SD. (DOCX 19 kb)

## Data Availability

The data used to support the findings of this study are included within the article.
