# Supplementary material for: Nicorandil Ameliorates Doxorubicin-Induced Cardiotoxicity in Rats, as Evaluated by 7 T Cardiovascular Magnetic Resonance Imaging
Source: Cardiovasc Drugs Ther. 2021 Sep 30;37(1):39–51. doi: 10.1007/s10557-021-07252-5 (PMC9834367; doi:10.1007/s10557-021-07252-5)
Supplement: Supplementary file 1 — Supplemental table. CMR in vivo data for all the time points represented by groups as means ± SD. (DOCX 19 kb) [file 10557_2021_7252_MOESM1_ESM.docx]

**Supplemental table.** CMR in vivo data for all the time points are represented by groups as the mean ± SD.

|  | **Control group** | | | | | **Nic group** | | | | |
| --- | --- | --- | --- | --- | --- | --- | --- | --- | --- | --- |
| **Week** | **LVEF(%)** | **LVM(g)** | **LVEDV(ml)** | **LVESV(ml)** | **T2(ms)** | **LVEF(%)** | **LVM(g)** | **LVEDV(ml)** | **LVESV(ml)** | **T2(ms)** |
| **0** | 69.8±1.84 | 0.31±0.01 | 0.27±0.01 | 0.08±0.01 | 22.59±1.59 | 69.91±1.7 | 0.31±0.01 | 0.28±0.01 | 0.08±0.01 | 22.55±1.34 |
| **1** | 69.55±2.05 | 0.36±0.02 | 0.32±0.03 | 0.09±0.01 | 22.13±2.09 | 69.24±3.37 | 0.36±0.02 | 0.32±0.02 | 0.09±0.01 | 22.54±1.11 |
| **2** | 69.41±2.76 | 0.46±0.03 | 0.41±0.04 | 0.13±0.01 | 23.02±1.79 | 70.13±2.64 | 0.46±0.03 | 0.41±0.03 | 0.13±0.01 | 22.42±0.6 |
| **3** | 69.12±3.94 | 0.48±0.03 | 0.44±0.02 | 0.15±0.01 | 22.22±1.85 | 69.74±3.58 | 0.49±0.02 | 0.44±0.36 | 0.14±0.01 | 21.93±1.18 |
| **4** | 70.13±2.87 | 0.53±0.38 | 0.49±0.02 | 0.16±0.01 | 22.18±1.51 | 69.74±2.71 | 0.53±0.35 | 0.47±0.05 | 0.16±0.01 | 22.27±1.91 |
| **5** | 69.81±2.84 | 0.5±0.02 | 0.47±0.02 | 0.17±0.01 | 23.2±1.33 | 70.49±5.06 | 0.5±0.01 | 0.47±0.02 | 0.16±0.01 | 23.09±1.76 |
| **6** | 70.03±2.98 | 0.5±0.03 | 0.48±0.03 | 0.17±0.01 | 23.17±2.42 | 71.05±4.4 | 0.5±0.02 | 0.47±0.02 | 0.17±0.01 | 22.52±1.45 |

|  | **Dox group** | | | | | **Dox+Nic group** | | | | |
| --- | --- | --- | --- | --- | --- | --- | --- | --- | --- | --- |
| **Week** | **LVEF(%)** | **LVM(g)** | **LVEDV(ml)** | **LVESV(ml)** | **T2(ms)** | **LVEF(%)** | **LVM(g)** | **LVEDV(ml)** | **LVESV(ml)** | **T2(ms)** |
| **0** | 69.8±1.71 | 0.31±0.02 | 0.27±0.02 | 0.08±0.01 | 22.68±1.66 | 69.18±1.03 | 0.32±0.02 | 0.29±0.03 | 0.08±0.01 | 22.46±0.71 |
| **1** | 68.85±1.54 | 0.35±0.01 | 0.3±0.03 | 0.09±0.01 | 23.7±1.76 | 69.58±2.78 | 0.36±0.02 | 0.31±0.02 | 0.09±0.01 | 22.6±1.28 |
| **2** | 69.17±3.2 | 0.47±0.01 | 0.42±0.03 | 0.13±0.01 | 27.84±0.95 | 68.85±2.17 | 0.47±0.01 | 0.44±0.02 | 0.14±0.01 | 24.6±0.6 |
| **3** | 67.85±1.64 | 0.5±0.01 | 0.46±0.03 | 0.16±0.01 | 23.5±1.71 | 68.1±1.45 | 0.5±0.01 | 0.45±0.04 | 0.16±0.01 | 23.64±2.6 |
| **4** | 64.51±1.56 | 0.50±0.01 | 0.44±0.02 | 0.17±0.01 | 22.31±0.89 | 67.97±3.4 | 0.55±0.01 | 0.47±0.02 | 0.17±0.01 | 22.9±1.64 |
| **5** | 60.88±2.4 | 0.49±0.02 | 0.44±0.02 | 0.18±0.01 | 21.69±1.11 | 65.84±2.59 | 0.51±0.05 | 0.50±0.03 | 0.17±0.01 | 22.5±0.9 |
| **6** | 53.31±1.74 | 0.5±0.01 | 0.42±0.01 | 0.18±0.01 | 21.52±2.4 | 63.92±2.42 | 0.52±0.03 | 0.47±0.04 | 0.17±0.01 | 21.78±1.81 |

|  | **Dox+DZ group** | | | | |
| --- | --- | --- | --- | --- | --- |
| **Week** | **LVEF(%)** | **LVM(g)** | **LVEDV(ml)** | **LVESV(ml)** | **T2(ms)** |
| **0** | 70.26±2.07 | 0.31±0.01 | 0.27±0.01 | 0.08±0.01 | 22.46±1.09 |
| **1** | 69.62±2.85 | 0.36±0.02 | 0.31±0.02 | 0.09±0.01 | 22.38±1.67 |
| **2** | 69.92±2.02 | 0.46±0.04 | 0.42±0.03 | 0.14±0.01 | 24.83±2.03 |
| **3** | 67.7±3.2 | 0.5±0.02 | 0.46±0.04 | 0.15±0.01 | 23.52±1.87 |
| **4** | 67.49±2.52 | 0.53±0.03 | 0.49±0.02 | 0.17±0.01 | 22.57±1.68 |
| **5** | 65.75±2.87 | 0.51±0.05 | 0.48±0.02 | 0.17±0.02 | 22.16±1.64 |
| **6** | 65.28±2.51 | 0.5±0.63 | 0.48±0.02 | 0.17±0.01 | 21.69±1.46 |
